# Force-Invariant Improved Feature Extraction Method for Upper-Limb Prostheses of Transradial Amputees

**DOI:** 10.3390/diagnostics11050843

**Published:** 2021-05-07

**Authors:** Md. Johirul Islam, Shamim Ahmad, Fahmida Haque, Mamun Bin Ibne Reaz, Mohammad Arif Sobhan Bhuiyan, Md. Rezaul Islam

**Affiliations:** 1 Department of Electrical and Electronic Engineering, University of Rajshahi, Rajshahi 6205, Bangladesh; johirulap@gmail.com (M.J.I.); rima@ru.ac.bd (M.R.I.); 2 Department of Physics, Rajshahi University of Engineering and Technology, Rajshahi 6204, Bangladesh; 3 Department of Computer Science and Engineering, University of Rajshahi, Rajshahi 6205, Bangladesh; shamim_cst@yahoo.com; 4 Department of Electrical, Electronic and Systems Engineering, Universiti Kebangsaan Malaysia—UKM, Bangi 43600, Malaysia; fahmida32@yahoo.com (F.H.); mamun@ukm.edu.my (M.B.I.R.); 5 Department of Electrical and Electronics Engineering, Xiamen University Malaysia, Bandar Sunsuria, Sepang 43900, Malaysia

**Keywords:** EMG pattern recognition, force-invariant features, nonlinear features, correlation coefficients

## Abstract

A force-invariant feature extraction method derives identical information for all force levels. However, the physiology of muscles makes it hard to extract this unique information. In this context, we propose an improved force-invariant feature extraction method based on nonlinear transformation of the power spectral moments, changes in amplitude, and the signal amplitude along with spatial correlation coefficients between channels. Nonlinear transformation balances the forces and increases the margin among the gestures. Additionally, the correlation coefficient between channels evaluates the amount of spatial correlation; however, it does not evaluate the strength of the electromyogram signal. To evaluate the robustness of the proposed method, we use the electromyogram dataset containing nine transradial amputees. In this study, the performance is evaluated using three classifiers with six existing feature extraction methods. The proposed feature extraction method yields a higher pattern recognition performance, and significant improvements in accuracy, sensitivity, specificity, precision, and F1 score are found. In addition, the proposed method requires comparatively less computational time and memory, which makes it more robust than other well-known feature extraction methods.

## 1. Introduction

Electromyography (EMG) measures the electrical activity of muscles, which possess information related to their movement [1,2]. Generally, two techniques are widely used for EMG signal acquisition: surface EMG and needle EMG [3]. Recently proposed noninvasive and contactless capacitive EMG is also very promising for the acquisition of EMG signals [4,5,6,7]. However, a feature extraction method evaluates the information indicating a unique movement. Consequently, EMG signals are widely used as a control strategy in myoelectric pattern recognition [8]. However, myoelectric prosthetic hand users are not satisfied with the performance and the degree of freedom of available prosthetic hand [9]. The performance of myoelectric pattern recognition is highly influenced by wrist orientation [10,11], arm positions [12,13], electrode shift [14,15,16], non-stationarity characteristics of the signal [17], mobility of subject [18], and muscle force variation [18,19,20,21]. Among these crucial parameters, force variation is one of the vital physiological behaviors of skeletal muscle, which plays a key role in varying the amplitude and frequency characteristics of the EMG signal [22,23]. Therefore, researchers tried to resolve the force variation problem in myoelectric pattern recognition.

Tkach et al. [24] studied the stability of EMG pattern recognition performance of eleven time-domain features with low and high force levels using linear discriminant analysis (LDA). They observed that the individual pattern recognition performance of each of the time-domain features degraded when the testing force level was not used in the training phase. In addition, they observed that the autoregression coefficient (AR) feature showed better performance with the variation in muscle force. The AR along with the root mean square (RMS) feature were reported by Huang et al. too [25].

Scheme et al. [20] investigated the problems associated with force variation on the EMG pattern recognition performance. In that study, they involved intact-limb subjects with ten hand movements. They collected EMG data for a wide range of force variation, i.e., ranging from 20% to 80% of the maximum voluntary contraction (MVC) with a step size of 10%. They observed a high error rate ranging from 32% to 45% with the LDA classifier; in that study, the LDA was trained with a single force level and was tested with all force levels. In their training scheme, a 50% training force level achieved the lowest error rate. However, the classifier improved its performance with an error rate of 16% when the classifier was trained with all force levels.

Al-Timemy et al. [19] proposed the time-dependent power spectrum descriptors (TDPSD) feature extraction method; it was based on an orientation between a set of spectral moments and a nonlinear map of the original EMG signal. In that study, they involved nine amputees to collect EMG data associated with three force levels; each amputee performed six hand gestures. In that study, the TDPSD achieved significant improvements from ≈6% to 8% in the averaged values of classification performance in comparison with that of well-known four feature extraction methods when the LDA classifier was trained with all force levels. Furthermore, Khushaba et al. [26] proposed the temporal-spatial descriptors (TSD), where they evaluated seven temporal features from a window and spatial correlation between channels, i.e., *C_x_-C_y_*. They evaluated the performance on five datasets, where amputees were involved in three datasets. TSD achieved a significant improvement: at least 8% in the averaged value of classification performances for all subjects.

Most of the authors proposed their feature extraction methods to improve force-invariant EMG pattern recognition performance, and they utilized multiple force levels for training purposes to achieve performance at a satisfactory level. However, an ideal force-invariant feature extraction method is such that a single force level is used for the training purpose but is capable of recognizing the gestures at the force level used in training and the gestures at other force levels [27]. Moreover, less time for feature extraction and smaller memory sizes are highly desired, so that the system is implementable in a microcontroller [28,29,30,31].

He et al. [27] proposed a feature extraction method based on discrete Fourier transform and muscle coordination. In this study, they involved intact-limb subjects with a specific location for electrode placement. The subjects performed eight gestures associated with three force levels, where low, medium, and high force levels were defined as 20%, 50%, and 80% of the MVC, respectively. Their proposed method achieved an improvement of 11% in an average performance in comparison with those of time-domain features. In addition, they achieved 91% force-invariant EMG pattern recognition performance when a medium force level was used for training purpose. However, the major constraint of this work is that it requires a specific electrode position on the forearm, which is quite hard to ensure for all amputees. A short overview of the different feature extraction methods is shown in Table 1.

In this context, we attempt to improve the force-invariant EMG pattern recognition performance of transradial amputees. It is more challenging than that for intact-limb subjects since the muscle structure of the amputee is not perfect as for intact-limb subject [35,36]. In this study, we propose an improved force-invariant feature extraction method. It is the extension of the pilot work of Khushaba et al. [26], where the authors used higher-order moments as a feature [13,19,26]; however, they did not use frequency information of the corresponding higher-order moments. However, Hudgin et al. [34] suggested that frequency information along with EMG signal strength obtain better performances. Therefore, to determine the higher-order spectral moments along with frequency information, we employ the time derivative of the signal [26]. Moreover, all considered features are nonlinearly transformed, which associates the EMG signal with a low force more discriminable than that of the high force level. Thus, this transformation balances the forces associated with different gestures and enhances the separation margin among those gestures. In addition to these nonlinear features, we consider the correlation coefficient (CC) for all channel pairs; it requires less computational time since only a single parameter is calculated instead of calculating all of the features, which are mentioned in [26]. An interesting salient characteristic of the CC is that it determines the correlation between channels placed on the underlying muscle groups except for the amplitude of the EMG signal, which is proportionally varied with respect to the muscle force level. Therefore, it is expected that the CC would perform well in force-invariant EMG pattern recognition performance.

In this study, we use an EMG dataset containing transradial amputees to evaluate force-invariant EMG pattern recognition performance when the proposed feature extraction method is used. In addition, we compare the performance and robustness between the proposed feature extraction method and the existing six well-known feature extraction methods with respect to three different classifiers.

The remainder of this paper is structured as follows. Section 2 describes the proposed feature extraction method, EMG dataset, and EMG pattern recognition method. Section 3 shows the force-invariant EMG pattern recognition performance, where the resulting performances are compared with those of other considered well-known feature extraction methods. Section 4 investigates the reasons behind the obtained improved performance, and Section 5 summarizes the overall experimental results.

## 2. Materials and Methods

### 2.1. The Proposed Feature Extraction Method

A discrete EMG signal can be expressed for window size *N* as x[iT], i=0,1,2,3,.....,N−1, with a sampling frequency of $f_{S}$ Hz, where $T=\frac{1}{f_{S}}$. However, x[iT] is also expressed as x[i]. Parseval Theorem in Equation (1) states that the sum of the square of a function is identical to the sum of the square of its Fourier transform.

(1) $$\sum_{i=0}^{N-1}x{\left[i\right]}^{2}=\frac{1}{N}\sum_{k=0}^{N-1}X[k]X^{*}[k]=\sum_{k=0}^{N-1}P[k]$$

where $X^{*}[k]$ is the conjugate of X[k] and P[k] is the corresponding power spectral density with a frequency index of k. The following equation relates the derivative of the time-domain signal to the frequency-domain signal.

(2) $$F[\Delta^{n}x[i]]=k^{n}X[k]$$

where *F* is the discrete Fourier transform operator and *n* is the order of derivative. Therefore, the proposed features using Equations (1) and (2) are as follows:

**Zero-order power spectrum (*P*_0_):** The zero-order power spectrum measures the signal strength in the frequency domain [13,19,26]. According to Equation (1), $P_{0}$ can be defined in the following way.

(3) $$P_{0}=\sum_{i=0}^{N-1}x{\left[i\right]}^{2}$$

**Second-**, **fourth-, and sixth-order power spectra (*P*_2_, *P*_4_, and *P*_6_):** Hjorth et al. [37] defined a second-order moment as the power of the signal. Therefore, according to Equation (2), it is defined as follows:

(4) $$P_{2}=\sum_{k=0}^{N-1}k^{2}P[k]=\frac{1}{N}\sum_{k=0}^{N-1}{\left[kX[k]\right]}^{2}=\sum_{i=0}^{N-1}{\left[\Delta x[i]\right]}^{2}$$

Therefore, the higher-order power spectrums are defined by repeating the process.

(5) $$P_{4}=\sum_{k=0}^{N-1}k^{4}P[k]=\frac{1}{N}\sum_{k=0}^{N-1}{\left[k^{2}X[k]\right]}^{2}=\sum_{i=0}^{N-1}{\left[\Delta^{2}x[i]\right]}^{2}$$

(6) $$P_{6}=\sum_{k=0}^{N-1}k^{6}P[k]=\frac{1}{N}\sum_{k=0}^{N-1}{\left[k^{3}X[k]\right]}^{2}=\sum_{i=0}^{N-1}{\left[\Delta^{3}x[i]\right]}^{2}$$

The odd-order power spectrums are zero. As a result, only effective even order power spectra $P_{2}$, $P_{4}$, and $P_{6}$ are considered.

**First- and second-order average amplitude change (*AC*_1_ and *AC*_2_):** Unlike in [34], the average of changes in amplitude denotes indirect frequency information. A higher change in amplitude implies higher frequency and vice versa.

(7) $$AC_{1}=\frac{1}{N-1}\sum_{i=0}^{N-1}\left|\Delta x\right|$$

(8) $$AC_{2}=\frac{1}{N-2}\sum_{i=0}^{N-1}\left|\Delta^{2}x\right|$$

**Mean Value (*MV*):** According to [34], the mean value represents the signal strength that can be defined mathematically,

(9) $$MV=\frac{1}{N}\sum_{i=0}^{N-1}\left|x[i]\right|$$

**Figure 1:**
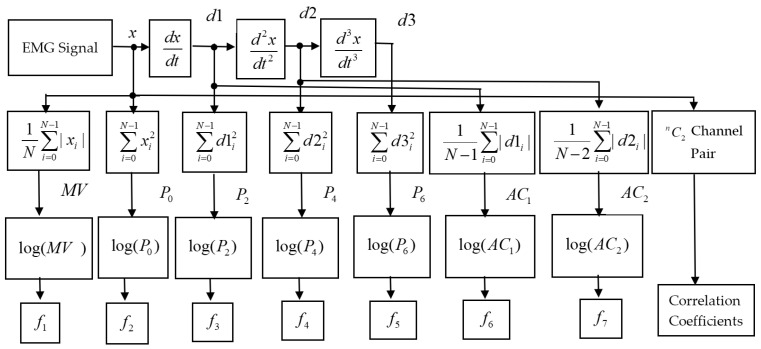
The block diagram of the proposed feature extraction procedure.

EMG pattern recognition performance varies with respect to force variation [19]. In addition, EMG signals, when their amplitude values are small, also suffer from the least separable margin among them. Some of the nonlinear functions, the square root, and logarithm were used, which were described in [13,31]. Besides these, we additionally employed the logarithm (log_e_x) on the seven extracted features to obtain the final features, $f_{1},f_{2},f_{3},f_{4},f_{5},f_{6},$ and $f_{7}$ as shown in Figure 1.

**Correlation coefficients:** The size of a motor unit and its firing rate change muscle force, which in turn play a role in varying the EMG signal’s amplitude and its frequency spectrum [22]. Consequently, the amplitude- and frequency-domain features extracted from that EMG signal also fluctuate. Naturally, these fluctuations of the features highly affect EMG pattern recognition performance [20,27]. However, this problem caused by the force variation can be minimized if the features are made force independent.

The CC statistically determines the strength and direction of a linear relationship between two variables. The most salient feature of CC is that it is independent of origin and the unit of measurement of the two considered variables. In the case of multichannel EMG signal acquisition, the CC between any two channels placed on the underlying muscles varies with respect to the gestures since active muscles are unique for each gesture. Additionally, the active muscles that change the strength of the EMG signal remain unchanged for all forces [27]. Therefore, it is expected that the CC is a force-independent feature. The linear correlation coefficient *ρ(x,y)* for the channels *x* and *y* is given by the following formula.

(10) $$\rho (x,y)=\frac{Cov(x,y)}{\sigma_{x}\sigma_{y}}=\frac{\sum_{i=0}^{N-1}\left(x_{i}-\bar{x}\right)(y_{i}-\bar{y})}{\sqrt{\sum_{i=0}^{N-1}{\left(x_{i}-\bar{x}\right)}^{2}}\sqrt{\sum_{i=0}^{N-1}{\left(y_{i}-y\right)}^{2}}}$$

Where $\bar{x,}$$\bar{y,}$ and N represent the mean of the channel x, the mean of the channel y, and the number of samples in a channel, respectively. If there exists n number of channels, then the number of channel pair is C2n, which is equal to the dimension of the correlation coefficient feature. The whole feature extraction procedure is as follows:

### 2.2. Description of EMG Dataset

**Figure 2:**
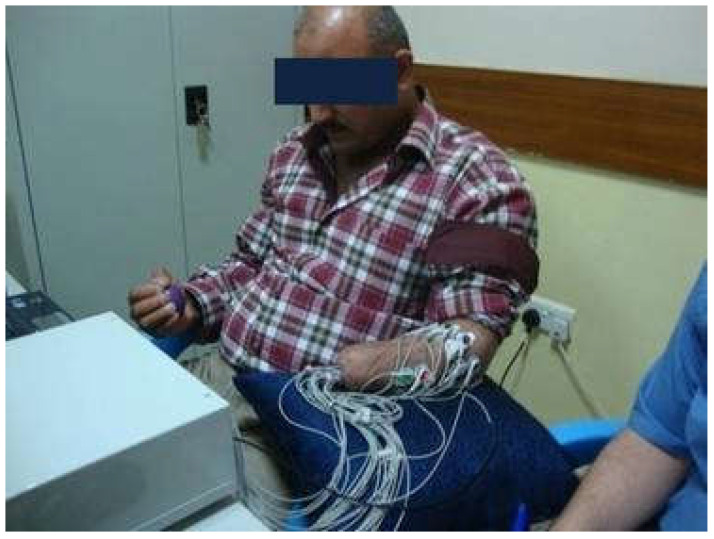
The position of electrodes for EMG data acquisition from an amputee. Source: Electromyogram (EMG) repository (rami-khushaba.com) (accessed on 07 May 2021).

The EMG dataset of transradial amputees was collected from the dedicated website of the second author [19]. The dataset contains nine transradial amputees, seven traumatic (TR1–TR7), and two congenital (CG1 and CG2) amputees, where each amputee was asked to perform six gestures during the process of data collection. The considered gestures were thumb flexion, index flexion, fine pinch, tripod grip, hook grip (hook or snap), and spherical grip (power). However, it was a very challenging task for transradial amputees to perform an imaginary gesture. Therefore, the amputees employed the support of their intact hand to perform an imaginary gesture. In addition to their intact hand, the amputees also used the LabVIEW (National Instruments, Ostin, TX, USA) software to observe the visual feedback for each channel. During this EMG data collection process, each amputee produced three force levels; those were defined as low, medium, and high. They maintained different force levels while watching real-time EMG signal displayed on the LabVIEW screen. However, each transradial amputee performed five to eight trials with a duration of 8 to 12 s. Thus, the total number of EMG signals collected from an amputee is equal to the product of the number of forces, gestures, and trials. In this EMG signal acquisition process, a custom-build EMG signal acquisition system was employed, where the EMG signal was sampled at 2000 Hz. Additionally, the Ag/AgCl electrode (Tyco healthcare, Germany) was used. In this data collection process, differential signal electrode pairs were placed around the forearm of the amputee and their ground electrode was placed on the elbow joint (Figure 2). In this dataset, the number of EMG signal channel varied (8 to 12) from one amputee to another depending on the remaining stump length; however, the first eight channels are common to all amputees and these electrodes were placed around their forearm only. Therefore, we employed data collected from these eight channels to evaluate the EMG pattern recognition [19]. In addition to the considered electrode position, we employed the first five trials for the evaluation of EMG pattern recognition performance; each trial collected at different times indicated the identical gesture. However, to maintain the 5-fold cross-validation described in Section 2.3, we considered the first five trials.

### 2.3. EMG Pattern Recognition 

In this study, for the performance analysis of EMG pattern recognition, we used the popular software MATLAB^®^ 2017a (Mathworks, Natick, MA, USA). An overlapped rectangular windowing scheme was used with a duration of 150 ms, and adjacent windows were overlapped with a duration of 50 ms [19]. The required average delay between successive prediction was 100+τ ms (τ is the required time for predicting a classifier); therefore, the processing time or average system delay was set within the acceptable limit of the real-time prosthetic hand [38]. Each window with a duration of 150 ms for the EMG signal was preprocessed using cascaded digital filters, where a high pass filter of 20 Hz, a low pass filter of 500 Hz, and a notch filter of 50 Hz were used to remove movement artefact [39], high-frequency noise [28], and power line artefact [40], respectively. In the feature extraction section, the proposed features, $f_{1},f_{2},f_{3},f_{4},f_{5},f_{6},f_{7}$, and CC, were evaluated with a feature dimension of 84 (number of features × number of channels + C2n correlation coefficients = 7 × 8 + 28 = 84). Therefore, we compared the proposed feature extraction method against six well-known feature extraction methods associated with three different force levels. These include the following:

TSD [26] describes seven features, the root squared zero-order, second-order, and fourth-order moments; sparseness; irregularity factor; coefficient of variation; and the Teager–Kaiser energy operator. Additionally, these seven features were evaluated from each difference between pairs of channels, i.e., $C_{x}-C_{y}$. Therefore, TSD provides 252 dimensional features (number of features × (number of channels + C2n pair of $C_{x}-C_{y}$) = 7 × (8 + 28) = 252).

TDPSD [19] defines six features that are extracted from the time-domain EMG signal. TDPSD features include the root squared zero-order, second-order, and fourth-order moments; the sparseness; the irregularity factor; and waveform length ratio. Hence, TDPSD provides a 48-dimensional feature space.

Wavelet features [32] includes the energy, variance, standard deviation, waveform length, and entropy computed from five levels of decomposition of the coefficients using the Symmlet-8 wavelet family. The wavelet feature dimension is 240 (number of features × (decomposition level + original) × number of channels = 5 × (5 + 1) × 8 = 240).

Du et al. [33] used the six time-domain features (TDF), which were the integral of EMG, waveform length, variance, zero-crossing, slope sign change, and the Wilson amplitude. Therefore, the dimension of the TDF is 48.

Huang et al. [25] used seven features, which were the six order of AR along with the RMS value (AR-RMS). It created a 56-dimensional feature space.

Hudgin et al. [34] defined five features, with four of them (TD) being very popular for myoelectric pattern recognition: the mean absolute value, waveform length, zero-crossing, and slope sign change. Therefore, these four features produce a 32-dimensional feature space.

To reduce the computational time, a higher dimensional feature space was reduced to c−1(c is the number of gestures) by using the spectral regression discriminant analysis [41]. In EMG pattern recognition, different classifiers are widely used. These are convolutional neural networks (CNNs) [42,43], artificial neural networks (ANNs) [1,44], linear discriminant analysis (LDAs) [45], support vector machines (SVMs) [46,47], and k-nearest neighbors (KNNs) [48,49]. Among these classifiers, the CNN provides better EMG recognition performance but requires a higher time for learning the model [50]. Therefore, we employed widely used classifiers: the LDA with quadratic function [20,51], the SVM with gaussian radian basis function [46], and the KNN with the number of neighbors equal to three [13]. In this performance evaluation, four trials from the first five were used as training data and the remaining one was used as testing data. Additionally, the process was repeated five times so that each of the trials was used as testing data, which is called 5-fold cross-validation. In this performance evaluation, the performance (*F*1 score) of each fold is found consistent with respect to other folds, which confirms that the data are not overfitted. However, the number of the training sample is equal to the product of the number of training force levels, training trials, gestures, and the number of samples per trial. Similarly, the number of testing sample is the product of the number of testing force levels, testing trials, gestures, and the samples per trial. In this dataset, the EMG signal duration varies from 8 to 12 s. Hence, the number of training and testing samples also varies slightly from one amputee to another. Finally, the EMG pattern recognition performance was measured in terms of accuracy, sensitivity, specificity, precision, and F1 score [52,53]. These parameters are evaluated as follows:

(11) $$Accuracy=\frac{TP+TN}{TP+TN+FP+FN}$$

(12) $$Sensitivity=\frac{TP}{TP+FN}$$

(13) $$Specificity=\frac{TN}{TN+FP}$$

(14) $$Precision=\frac{TP}{TP+FP}$$

(15) $$F1Score=\frac{2\times Precision\times Sensitivity}{Precision+Sensitivity}$$

where *TP, TN, FP,* and *FN* represent true positive, true negative, false positive, and false negative value values, respectively.

### 2.4. EMG Pattern Recognition Performance with Training Strategies of Various Force Level

In daily life, we frequently change muscle forces as required for every movement. Recent studies [19,20,27] illustrated the effect of training strategies with respect to various force levels on EMG pattern recognition performance. Therefore, we study some training and testing schemes for the proposed feature extraction method and considered well-known feature extraction methods

Case 1: Training and testing the classifiers with the same force level.

Case 2: Training the classifiers with a single force level at a time and testing the classifiers with all three force levels.

Case 3: Training the classifiers with any two force levels at a time and testing the classifiers with all three force levels.

Case 4: Training the classifiers with all three force levels and testing the classifiers with all three force levels.

### 2.5. Statistical Test

To determine the significant difference between the proposed method and other methods, the Bonferroni-corrected Analysis of Variance (ANOVA) test is utilized with a significant level of 0.05. The obtained *p*-values below 0.05 imply that the performances of the proposed method are significantly different. In this study, the EMG pattern recognition performances of nine amputees for each training case (i.e., Case 1, Case 2, and Case 3) are concatenated to construct a 27-dimensional vector (9 amputees × 3 training schemes for each case), and then, the Bonferroni-corrected ANOVA is performed. Additionally, only ANOVA is performed in Case 4, where the number of training case is one.

### 2.6. RES Index

To evaluate the clustering performance of a feature or a feature extraction method, the *RES* (ratio of Euclidean distance to standard deviation) index is employed. The higher *RES index* specifies a higher separation margin among the classes and vice versa. The *RES index* can be evaluated as follows [54]:

(16) $$RES\;Index=\frac{\bar{ED}}{\bar{\sigma }}$$

where $\bar{ED}$ is the Euclidean distance between gesture *p* and *q;* it is defined mathematically,

(17) $$\bar{ED}=\frac{2}{K(K-1)}\sum_{p=1}^{K-1}\sum_{q=p+1}^{K}\sqrt{{\left(m_{1p}-m_{1q}\right)}^{2}+{\left(m_{2p}-m_{2q}\right)}^{2}}$$

where *m* and *K* denote the mean value of a feature and the total number of gestures. Dispersion of cluster *p* and *q* is given by

(18) $$\bar{\sigma }=\frac{1}{IK}\sum_{i=1}^{I}\sum_{k=1}^{K}S_{ik}$$

where *I* is the size of the feature vector.

## 3. Results

### 3.1. Signal Observation

**Figure 3:**
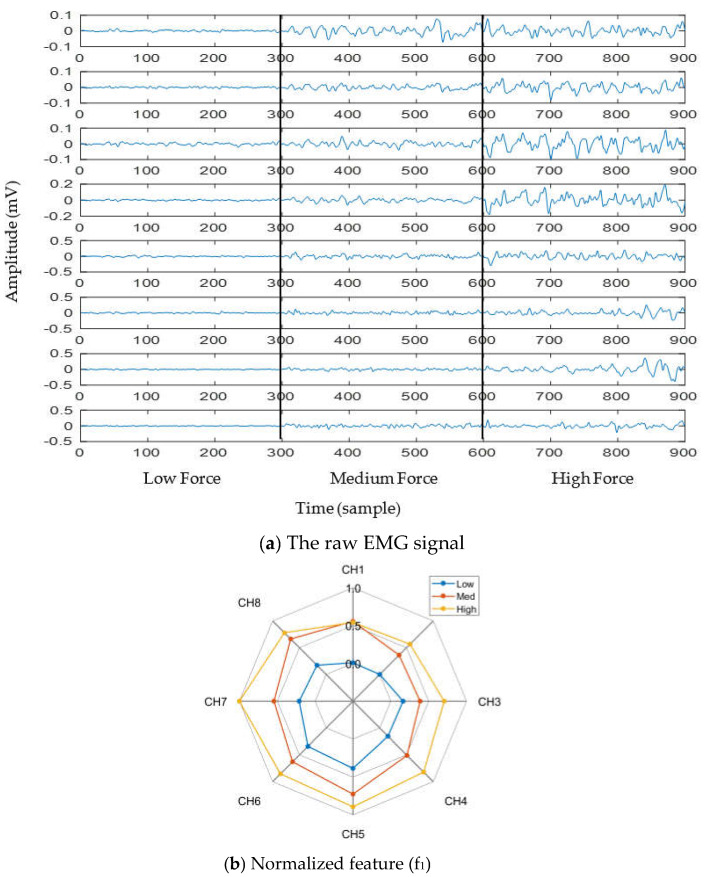
The impact of muscle force variation on a gesture (thumb flexion), where (**a**) presents the raw EMG signal and (**b**) presents normalized feature (f_1_).

To observe the impact of muscle force variation on a gesture, we considered thumb flexion hand gesture. Figure 3a shows the raw EMG signal for three muscle force levels (low, medium, and high) considering a window size of 150 ms. In addition to the raw EMG signal, one feature (f_1_) was calculated and is shown in the spider plot (Figure 3b). Both figures indicate that the EMG signal strength increases with respect to the increase in muscle force level. Additionally, it is noticed from Figure 3b that, although the strength of the EMG signal increases with respect to the increase in muscle force level, the muscle activation pattern obtained throughout the channels is almost unique for all force levels.

### 3.2. Impact of Nonlinear Transformation

**Figure 4:**
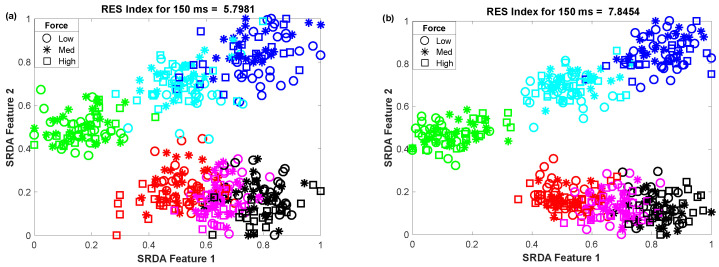
The impact of the nonlinear transformation of seven features on a 2D-feature space: (**a**) original feature space and (**b**) nonlinearly transformed feature space.

The impact of the nonlinear transformation (logarithm) on the 2D-feature space is shown in the scatter plot (Figure 4). In this scatter plot, we employed 25 sample points for each gesture from the dataset of amputee 1. Thus, the total number of sample points on the scatter plot is equal to 450 (muscle force levels × gesture × number of sample points for each movement = 3 × 6 × 25). The left (Figure 4a) and right (Figure 4b) scatter plots indicate the original features ($MV,P_{0},P_{2},P_{4},P_{6},AC_{1},AC_{2},$ and CC) and nonlinearly transformed features ($f_{1},f_{2},f_{3},f_{4},f_{5},f_{6},f_{7}$, and CC). In these figures, each color indicates a gesture. First, the 84-dimensional feature space for each force level is reduced to a 5-dimensional feature space using the SRDA. Thereafter, among this 5-dimensional feature space, the first two were normalized and were used for these scatter plots. The figures show that there is an almost unique muscle activation pattern among the gestures associated with all force levels. Additionally, the logarithm discriminates more for low amplitude values and discriminates less for high amplitude values. Figure 4b shows a higher *RES index* than Figure 4a, which means that the margin among the gestures is increased. In addition to an improvement in separation margin, the nonlinear transformation also has a more compact cluster among the forces for each gesture.

### 3.3. The Impact of Window Length on Clustering Performance

**Figure 5:**
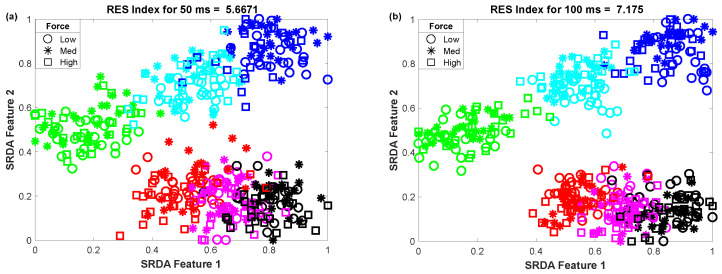

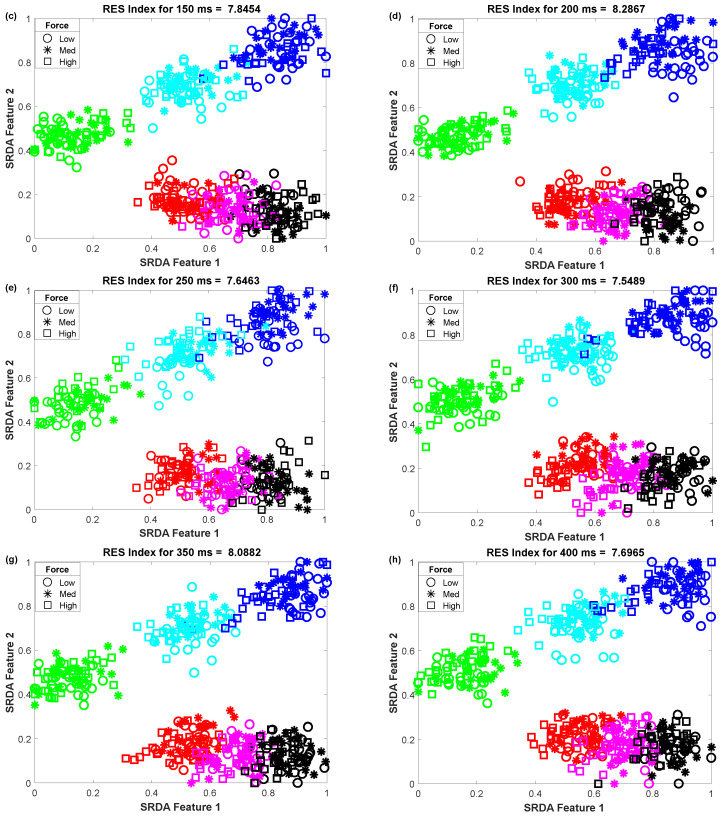
The impact of window length on clustering performance, where (**a**–**h**) stand for window lengths of 50 ms, 100 ms, 150 ms, 200 ms, 250 ms, 300 ms, 350 ms, and 400 ms, respectively.

To determine the impact of variable window length on clustering performance, we vary the window length from 50 ms to 400 ms with an equal interval of 50 ms. Then, we observed the scatter plot and the *RES index* simultaneously, which is shown in Figure 5. In this performance evaluation, the scatter plot visualizes the clustering performance and the separation margin among the gestures, and the *RES index* indicates their quantitative value. However, to evaluate the performance, we employed 25 sample points for each gesture from the dataset of amputee 1. Thus, the total number of sample points on the scatter plot is equal to 450 (muscle force levels × gesture × number of sample points for each movement = 3 × 6 × 25). First, 84-dimensional feature space associated with each force level was reduced to a 5-dimensional feature space using the SRDA. Thereafter, among this 5-dimensional feature space, the first two features (SRDA feature 1 and SRDA feature 2) were normalized and were used in scatter plots. The experimental results shown in Figure 5 indicate that the clustering performance (*RES index*) decreases with respect to the decrease of window length. It is also observed that there are some fluctuations in performance (*RES index*) when the window length is higher than 200 ms. The stochastic nature of the EMG signal may be a reason behind this fluctuation of clustering performance.

### 3.4. Training and Testing the Classifiers with Same Force Level (Case 1)

**Figure 6:**
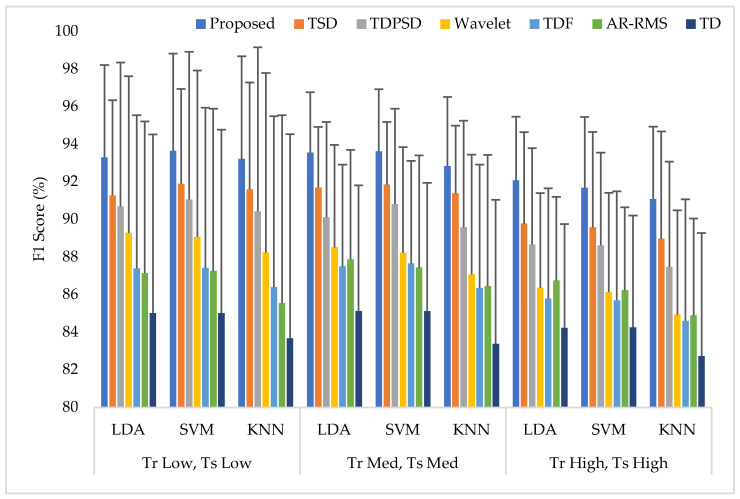
The EMG pattern recognition performances when the training and testing forces are the same, where Tr and Ts indicate training and testing, respectively.

Training and testing the classifiers with the same force level is a common strategy found in many studies. In this training and testing scheme, the average EMG pattern recognition performances across nine transradial amputees were evaluated by accuracy, sensitivity, specificity, precision, and F1 score, which are shown in Appendix A (Table A1). In addition, the performances are also graphically shown in Figure 6 using the F1 score, since the F1 score is a combined outcome of sensitivity and precision. The experimental results imply that the proposed feature extraction method yields the highest EMG pattern recognition performance in terms of all performance evaluating parameters compared to those of the considered existing feature extraction methods. In this comparison, the recently proposed TSD yields the second-highest EMG pattern recognition performance. The proposed feature extraction method improves the accuracy, sensitivity, specificity, precision, and the F1 score by 0.58, 1.73, 0.32, 1.42, and 1.77, respectively, when the SVM classifier is trained and tested with a medium force level. In addition, the proposed feature extraction method shows a consistency in the performance improvement when the LDA and the KNN are used. Moreover, the significant difference between the proposed feature extraction method and each of the existing feature extraction methods is also confirmed by the Bonferroni-corrected ANOVA. The obtained highest *p*-value is $1.55\times 10^{-4}$ considering each of the performance-evaluating parameters with each classifier, which strongly indicate that the performance achieved by the proposed feature extraction method is significantly different from those of the other methods.

### 3.5. Training the Classifiers with a Single Force Level at a Time and Testing the Classifiers with All Three Force Levels (Case 2) 

**Figure 7:**
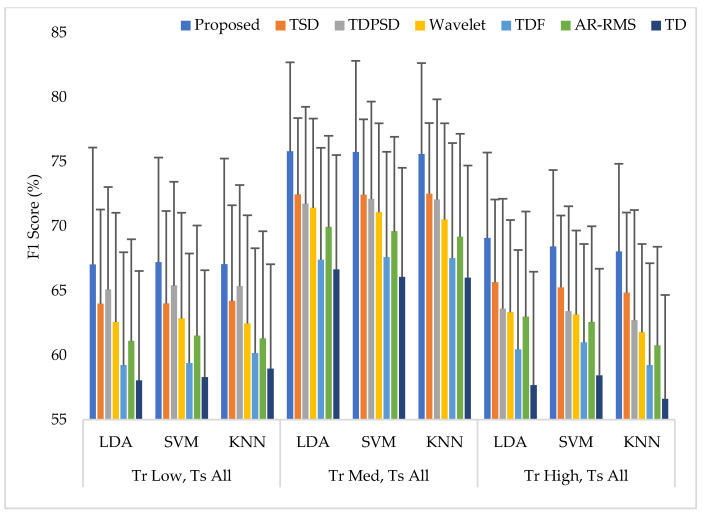
The EMG pattern recognition performances when training the classifiers with a single force level and testing with three force levels, where Tr and Ts indicate training and testing, respectively.

In this scheme (Case 2), the classifiers were trained with a single force level and then those were tested with that known force level used in training along with two other unknown force levels. The average performances for all performance evaluating parameters with standard deviation across nine amputees are represented in Appendix A (Table A2). The summary of Table A2 is also graphically shown in Figure 7, where the F1 score was employed only for simplicity. The experimental results show that unknown forces degrade the EMG pattern recognition performance compared to those obtained in Case 1. However, an interesting finding is that the classifiers can predict the unknown force levels as being low and high more effectively when the classifiers are trained with a medium force level. Additionally, in this single force level training scheme, the LDA and SVM classifiers yield almost the same EMG pattern recognition performances, which are slightly better than those obtained from the KNN classifier. However, even in the worst case, the proposed feature extraction method yields the highest EMG pattern recognition performance considering each performance evaluating parameters compared to those of other feature extraction methods. In the best case, when a medium force level training scheme is used, the proposed feature extraction method improves the accuracy, sensitivity, specificity, precision, and the *F*1 score by 1.12, 3.35, 0.67, 2.86, and 3.30, respectively, when the proposed method is compared with those of the TSD and the SVM classifier. Therefore, the obtained *p*-values between the proposed method and the other methods considering each classifier are very small, and its values are smaller than $9.27\times 10^{-4}$, which ensures a significant improvement by the proposed feature extraction method.

### 3.6. Training the Classifiers with Any Two Force Levels at a Time and Testing the Classifiers with All Three Force Levels (Case 3)

**Figure 8:**
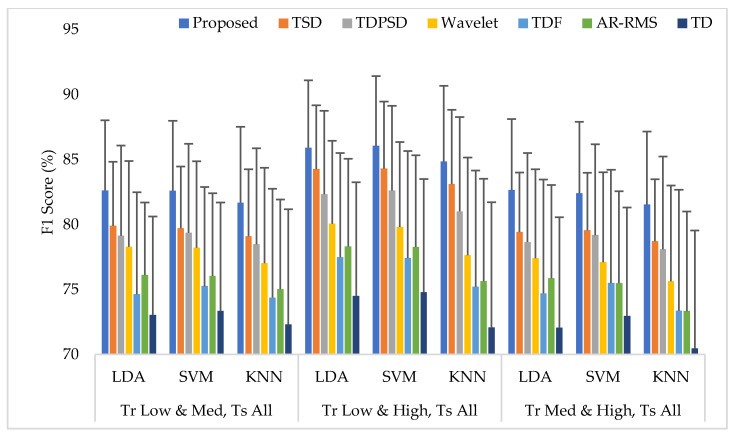
The EMG pattern recognition performances when the training forces are two and the testing forces are three, where Tr and Ts indicate training and testing, respectively.

The average EMG pattern recognition performances in terms of accuracy, sensitivity, specificity, precision, and F1 score for the proposed feature extraction method and those of the other well-known methods are shown in Appendix A (Table A3). In this case, the classifiers are trained with any two force levels and tested with all force levels. The EMG pattern recognition performances for different training pair of forces are graphically shown in Figure 8, where only a single parameter, the F1 score, is used for simplicity. The experimental results imply that, when the number of training force level is increased, the classifiers improve their pattern recognition performance in recognizing two known force levels used in training and an unknown force level. In this training case, we achieved an improvement in the F1 score by about 10% compared to that of Case 1. In this study, the proposed feature extraction method improves the accuracy, sensitivity, specificity, precision, and the F1 score by 0.57, 1.73, 0.36, 1.66, and 1.74, respectively, when the SVM classifier is trained with low and high force levels and is tested with all force levels. In addition, the obtained *p*-values between the proposed feature extraction method and each of the existing feature extraction methods considering each classifier are very low and the values are lower than $4.50\times 10^{-6}$, which shows a significant performance improvement by the proposed feature extraction method.

### 3.7. Training the Classifiers with all Three Force Levels and Testing the Classifiers with All Three Force Levels (Case 4)

**Figure 9:**
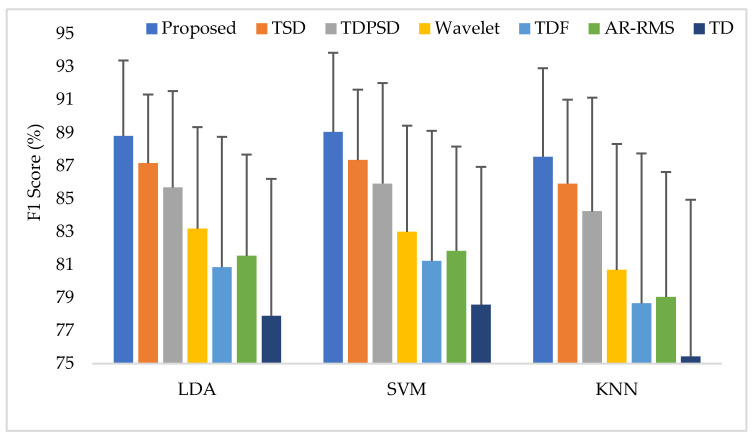
The average performances when the classifiers are trained and tested with three forces.

In this case, all force levels were used to train and test the classifiers. Then, the EMG pattern recognition performances in terms of accuracy, sensitivity, specificity, precision, and the F1 score were evaluated for all considered feature extraction methods, which are shown in Appendix A (Table A4). The EMG pattern recognition performances are also graphically shown in Figure 9 using the F1 score only. Following the previous trend, the proposed feature extraction method improves the accuracy, sensitivity, specificity, precision, and the F1 score by 0.57, 1.70, 0.33, 1.53, and 1.70, respectively, compared with those obtained from the TSD using the SVM classifier. In this study, the proposed method yields the highest F1 score of 89.06% with the SVM classifier. Therefore, ANOVA is performed between the proposed feature extraction method and each of the existing feature extraction methods for each classifier. The obtained *p*-values are very small, and the values are smaller than $1.21\times 10^{-2}$ considering all of the cases, which confirms the significant performance improvement by the proposed feature extraction method.

**Figure 10:**
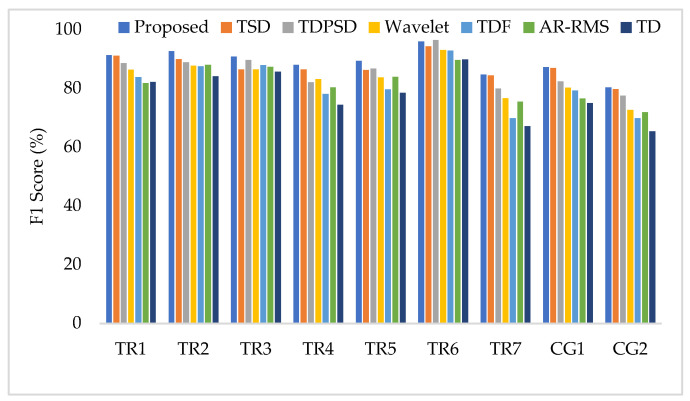
The amputee-wise performance when the SVM is trained and tested with three forces.

To compare the amputee-wise performance among all considered feature extraction methods, we used the SVM classifier only since it provides better performance in most cases. The obtained results shown in Figure 10 implies that the proposed feature extraction method yields the highest performance (F1 score) in most of the amputees (except for TR6). However, in some amputees (TR1, TR7, and CG1), TSD yields a performance similar to that of the proposed feature extraction method.

### 3.8. Computational Time and Memory Size

**Figure 11:**
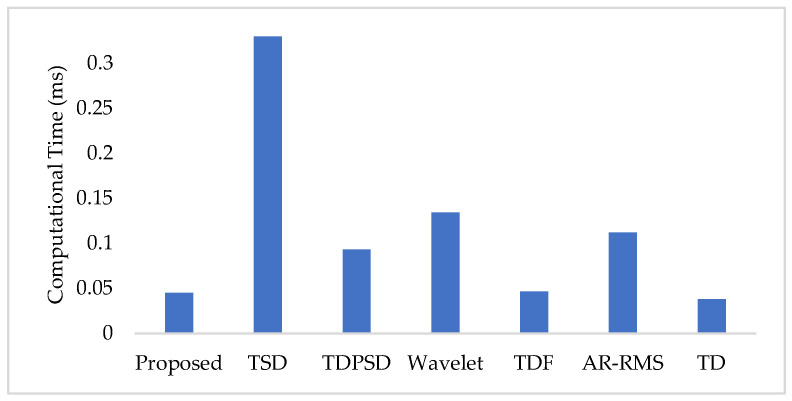
The feature extraction time for different feature extraction methods.

**Figure 12:**
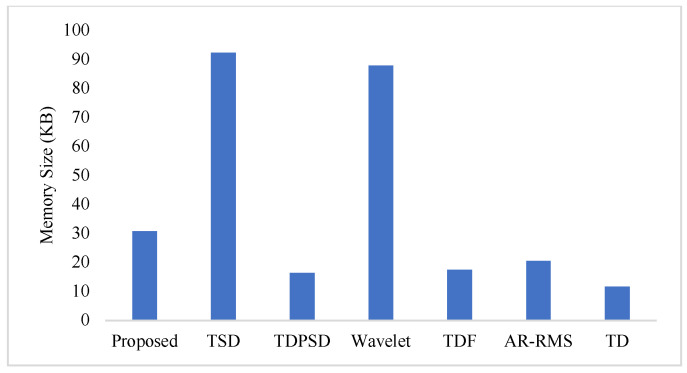
The memory size for different feature extraction methods.

To measure the computational load for each feature extraction method, we considered computational time and memory size [30,31]. We measured the computational time of each method using an Intel Core i3-7100U CPU with 2.40 GHz processor and 8 GB RAM; we used the MATLAB^®^ 2017a. The recorded computational times shown in Figure 11 demonstrate that the proposed feature extraction method requires the lowest time except for the TD. However, we know that TD offers a very low performance compared to those of the proposed method in all the cases. In addition to the computational time, we also computed the memory size used by each of the feature extraction methods; we used the MATLAB^®^ 2017a function (*whos*) for this purpose. Figure 12 shows that the proposed feature extraction method requires less memory than those required by TSD and Wavelet. Although TDPSD, TDF, AR-RMS, and TD require less memory than that required by the proposed feature extraction method, their EMG pattern recognition performances are lower than those of the proposed method. Therefore, we claim that the proposed feature extraction method is faster and requires less or compatible memory when recognition performance is taken into account. Therefore, the proposed method is suitable for real-time operation.

## 4. Discussion

Muscle force variation is a frequently used scenario in daily life. The amount of muscle force for a particular activity is set by the central nervous system (CNS), which is trained from our daily activities since childhood [27,55]. During muscle force variation, the CNS varies the time- and frequency-domain characteristics of the EMG signal, which in turn drastically varies the features that become unsuitable to achieve force-invariant EMG pattern recognition performance. Thus far, it is found that the EMG pattern recognition performance is significantly degraded when unknown force levels are used for testing [21,22,23]. The problem becomes more challenging when we consider amputees rather than intact-limb subjects [35,36].

In this study, we propose an improved force-invariant feature extraction method considering seven nonlinear features along with the CC, which is validated over nine transradial amputees and is compared with those of the six existing feature extraction methods considered. The proposed feature extraction method is an extension of the TSD and the TD [26,34], but it provides improved force-invariant EMG pattern recognition performance compared to those of the original works. In addition, the proposed feature extraction method requires less computational time than those required by other feature extraction methods except for the TD [19,25,26,32,33]. TD requires slightly less computational time than that of the proposed method, but the performance of the TD is very low and it cannot meet the criteria of a satisfactory performance [20]. In addition, the proposed feature extraction method also requires comparatively less memory than that required by the TSD and the Wavelet. Therefore, the proposed feature extraction method may be implemented using a microcontroller [30,31].

The proposed feature extraction method provides improved force-invariant pattern recognition performance due to the use of the higher-order indirect frequency information along with its moments, the nonlinear transformation of signals and the CC. The indirect frequency information of the higher-order differential signal is an important issue since the differential signal makes a nonlinear variation in frequency-domain, which in turn emphasizes the high-frequency EMG signal. Additionally, the nonlinear transformation balances the forces and enhances the separation margin among gestures. Finally, the CC measures the correlation between any two EMG channels, which has a great contribution to the improved force-invariant EMG pattern recognition obtained. The salient feature of CC is that it does not depend on the signal strength of each channel; in fact, it depends on the activity of the underlying active muscle. Hence, the CC values are varied with respect to the gesture only.

It is a challenging task to train the classifier by employing all possible force levels for each gesture such as how we utilize various force levels for each gesture in our daily activities [27]. In this context, the force-invariant EMG pattern recognition performance of our proposed feature extraction method shows good performance when we test the classifier with the gestures of an unknown force level (Case 2 and Case 3). The experimental results reveal that the proposed feature extraction method performs better in force-invariant EMG pattern recognition (Case 2 and Case 3) in terms of all performance evaluating parameters, i.e., accuracy, sensitivity, specificity, precision, and the F1 score. However, in this study, it is also observed that the proposed force-invariant feature extraction method does not yield EMG pattern recognition performance at a satisfactory level; this is due to the deformed structure of muscle of amputees and the lack of their proper training [35,36]. Therefore, the classifier is trained and tested with all force levels (Case 4); however, the robust character of the proposed feature extraction method is that it also performs better than those of existing feature extraction methods. In addition, the proposed feature extraction method performs better for regular EMG pattern recognition performance, when the classifier is trained and tested with one force levels (Case 1). In this training strategy, the performance obtained is much better (about 3 to 4 in the F1 score) than that of the classifier when trained and tested with all force levels (Case 4). The possible reason for degraded performance in Case 4 may be that the muscle activation pattern for each gesture of transradial amputees is not unique and is repetitive; that means that it does not follow the same manner among the force levels. Therefore, an amputee should be trained properly to achieve an improved EMG pattern recognition performance with respect to various force levels.

In this study, the performance of the proposed feature extraction method is evaluated with the LDA, SVM, and KNN classifiers with different training and testing cases. In all cases, the proposed feature extraction method shows consistently improved performance compared to those of existing feature extraction methods, which proves its robustness. Moreover, the lowest *p*-values between the proposed method and each of the methods also demonstrate the statistical significance of the experimental results. In this research, the LDA and the SVM classifiers show almost equal performances, which are slightly better than that for the KNN. However, the SVM classifier yields the highest F1 score of 89.06% with our proposed feature extraction method when the classifier is trained with three forces. The achieved performance is much better than the original work of the TDPSD, the TSD, and the recently proposed fractal feature set [19,26,56].

In this study, it is also observed that the classifiers yield the highest EMG pattern recognition performance when only medium force level, and combined low and high force levels are used to train the classifiers. It reveals that adjacent forces are highly interrelated, which may privilege the classifier for achieving the highest EMG pattern recognition performance. Therefore, it is suggested to train the classifier with such force levels that each testing force level is highly interrelated.

Another important point to note is that the traumatic amputees provide slightly better EMG pattern recognition performance than congenital amputees since they had intact limbs before the trauma occurred, and for this reason, they have better control over their muscle. However, regardless of the type of amputee, the proposed feature extraction method is promised to provide the highest or very close to the highest performance in all type of amputees.

In this study, we compared our proposed feature extraction method offline with respect to standard datasets collected from [19]. Real-time analysis with other amputees will be performed in future work.

## 5. Conclusions

In this research, a new time-domain feature extraction method is proposed to obtain improved force-invariant EMG pattern recognition performance. The proposed feature extraction method improves the performance across nine transradial amputees in terms of accuracy, sensitivity, specificity, precision, and F1 score. In addition to improved performance, it requires relatively less computational time and memory than others. In this study, the recently proposed method, TSD, provides the second-best performance after the proposed method, but it requires too much processing time and memory due to its high dimensional feature space. Moreover, Bonferroni-corrected ANOVA implies significant differences between the proposed method and the other methods. Therefore, the proposed feature extraction method is the best option to obtain improved force-invariant myoelectric pattern recognition using a microcontroller.

## Figures and Tables

**Table 1:** Different feature extraction methods.

Paper	Subject Type	Muscle Force Level	Feature	Classifier	Training Force	Accuracy (%)	Comment
Tkach et al. [24]	Intact	Low and high	Mean absolute value, zero crossings, slope sign change, waveform length, Wilson amplitude, variance, v-order, log detector, EMG histogram, AR, and cepstrum coefficients.	LDA	Low and high	82 with AR	Time-domain features are not stable with muscle force variation.
Huang et al. [25]	Intact	---	Mean absolute value, zero crossings, slope sign change, waveform length, AR, and RMS	Gaussian mixture model	---	96 AR + RMS	AR and RMS can be grouped for better EMG pattern recognition performance.
Scheme et al. [20]	Intact	20% to 80% of MVC at 10% interval	Time-domain features	LDA	20% to 80%	84	Time-domain features are not reliable with muscle force variation.
Al-Timemy et al. [19]	Amputee	Low, medium, and high	TDPSD includes root squared zero-order, second-order, and fourth-order moments; sparseness; irregularity factor; and waveform length ratio	LDA	All	90	TDPSD improves the performance with muscle force variation.
Khushaba et al. [26]	Intact and amputee	---	TSD, which includes root squared zero-order, second-order, and fourth-order moments; sparseness; irregularity factor; coefficient of variation; and Teager–Kaiser energy operator	LDA	---	99 (128 channel EMG)	TSD improves the EMG pattern recognition performance
He et al. [27]	Intact	Low, medium, and high	Global normalized discrete Fourier transform-based features	LDA	Medium	91	Force-invariant EMG pattern recognition performance is satisfactory, but the electrode position is specific.
Khushaba et al. [32]	Intact (driver drowsiness detection)	---	Symmlet-8 decomposition-based Wavelet features including energy, variance, standard deviation, waveform length, and entropy	LDA	---	97	Performance is better in another field, so the features may be applicable for force-invariant EMG pattern recognition.
Du et al. [33]	Intact	---	Time-domain features (TDF) including the integral of EMG, waveform length, variance, zero-crossing, slope sign change, and Wilson amplitude	Grey relational analysis	---	96	Performance is better, so these features may be utilized for force-invariant EMG pattern recognition.
Hudgin et al. [34]	Intact and amputee	---	Mean absolute value, mean absolute value slope, zero crossings, slope sign change, and waveform length	Neural Network	---	91.2 for intact subject and 85.5 for amputee	Performance is not satisfactory for amputees, but the features are fundamental.

## Data Availability

The EMG dataset is publicly available on the website of Rami N. Khushaba (https://www.rami-khushaba.com/electromyogram-emg-repository.html accessed on 7 May 2021).

## Appendix A. The EMG Pattern Recognition Performance for Different Training and Testing Cases

**Table A1:** The EMG pattern recognition performances when the classifiers are trained and tested with the same force level.

	Parameter	Classifier	Proposed	TSD	TDPSD	Wavelet	TDF	AR-RMS	TD
Training and testing with low force	Accuracy	LDA	97.86 ± 1.59	97.20 ± 1.65	96.97 ± 2.50	96.49 ± 2.73	95.88 ± 2.71	95.81 ± 2.69	95.17 ± 3.10
		SVM	97.93 ± 1.70	97.36 ± 1.67	97.06 ± 2.57	96.41 ± 2.91	95.88 ± 2.83	95.80 ± 2.88	95.11 ± 3.19
		KNN	97.78 ± 1.78	97.26 ± 1.86	96.87 ± 2.81	96.13 ± 3.13	95.55 ± 3.00	95.27 ± 3.28	94.69 ± 3.52
	Sensitivity	LDA	93.57 ± 4.77	91.60 ± 4.96	90.91 ± 7.50	89.46 ± 8.20	87.65 ± 8.14	87.43 ± 8.06	85.52 ± 9.31
		SVM	93.80 ± 5.09	92.09 ± 5.01	91.19 ± 7.72	89.22 ± 8.74	87.64 ± 8.48	87.39 ± 8.65	85.34 ± 9.56
		KNN	93.34 ± 5.35	91.79 ± 5.59	90.60 ± 8.44	88.40 ± 9.39	86.64 ± 8.99	85.80 ± 9.84	84.06 ± 10.55
	Specificity	LDA	98.71 ± 0.92	98.32 ± 0.96	98.21 ± 1.45	97.90 ± 1.60	97.56 ± 1.50	97.47 ± 1.59	97.14 ± 1.74
		SVM	98.75 ± 1.00	98.42 ± 0.98	98.27 ± 1.52	97.86 ± 1.72	97.56 ± 1.62	97.46 ± 1.74	97.12 ± 1.79
		KNN	98.65 ± 1.08	98.33 ± 1.12	98.12 ± 1.71	97.66 ± 1.89	97.32 ± 1.78	97.11 ± 2.04	96.82 ± 2.06
	Precision	LDA	94.48 ± 4.18	92.47 ± 4.42	91.86 ± 6.83	90.52 ± 7.58	88.95 ± 7.33	88.81 ± 7.62	87.07 ± 8.48
		SVM	94.46 ± 4.41	93.03 ± 4.62	92.09 ± 7.04	90.12 ± 8.14	88.83 ± 7.85	88.56 ± 8.08	86.91 ± 8.73
		KNN	93.95 ± 4.78	92.68 ± 5.17	91.43 ± 7.97	89.29 ± 8.89	87.69 ± 8.60	86.91 ± 9.54	85.47 ± 10.29
	F1 Score	LDA	93.30 ± 4.91	91.28 ± 5.06	90.69 ± 7.65	89.29 ± 8.32	87.39 ± 8.15	87.14 ± 8.07	85.01 ± 9.51
		SVM	93.64 ± 5.18	91.89 ± 5.05	91.05 ± 7.87	89.07 ± 8.85	87.42 ± 8.52	87.25 ± 8.64	85.01 ± 9.76
		KNN	93.22 ± 5.46	91.60 ± 5.69	90.44 ± 8.72	88.24 ± 9.55	86.39 ± 9.10	85.55 ± 10.0	83.67 ± 10.86
***Training and testing with medium force***	Accuracy	LDA	97.89 ± 1.05	97.30 ± 1.03	96.75 ± 1.68	96.21 ± 1.80	95.91 ± 1.79	96.00 ± 1.92	95.13 ± 2.21
		SVM	97.91 ± 1.09	97.33 ± 1.06	96.96 ± 1.68	96.12 ± 1.85	95.95 ± 1.8	95.85 ± 1.98	95.10 ± 2.25
		KNN	97.65 ± 1.20	97.17 ± 1.15	96.56 ± 1.87	95.75 ± 2.09	95.53 ± 2.17	95.53 ± 2.29	94.55 ± 2.52
	Sensitivity	LDA	93.66 ± 3.16	91.90 ± 3.09	90.25 ± 5.03	88.62 ± 5.40	87.72 ± 5.38	88.00 ± 5.77	85.40 ± 6.62
		SVM	93.72 ± 3.27	91.99 ± 3.19	90.87 ± 5.05	88.36 ± 5.55	87.84 ± 5.40	87.55 ± 5.94	85.31 ± 6.76
		KNN	92.96 ± 3.61	91.52 ± 3.45	89.68 ± 5.61	87.24 ± 6.28	86.59 ± 6.50	86.58 ± 6.87	83.66 ± 7.57
	Specificity	LDA	98.82 ± 0.64	98.50 ± 0.61	98.17 ± 0.99	97.82 ± 1.09	97.62 ± 1.08	97.71 ± 1.13	97.18 ± 1.32
		SVM	98.81 ± 0.65	98.49 ± 0.63	98.29 ± 0.99	97.76 ± 1.12	97.61 ± 1.10	97.61 ± 1.16	97.14 ± 1.36
		KNN	98.66 ± 0.72	98.41 ± 0.69	98.05 ± 1.09	97.53 ± 1.26	97.36 ± 1.32	97.42 ± 1.36	96.79 ± 1.53
	Precision	LDA	94.25 ± 3.16	92.87 ± 3.08	91.24 ± 4.97	89.64 ± 5.43	88.88 ± 5.28	88.83 ± 5.76	86.77 ± 6.29
		SVM	94.17 ± 3.21	92.75 ± 3.16	91.62 ± 4.96	89.33 ± 5.55	88.86 ± 5.35	88.49 ± 5.88	86.68 ± 6.57
		KNN	93.45 ± 3.52	92.26 ± 3.45	90.51 ± 5.52	88.08 ± 6.26	87.57 ± 6.39	87.38 ± 6.90	84.97 ± 7.40
	F1 Score	LDA	93.55 ± 3.21	91.70 ± 3.22	90.11 ± 5.07	88.51 ± 5.45	87.49 ± 5.42	87.87 ± 5.83	85.12 ± 6.69
		SVM	93.62 ± 3.31	91.85 ± 3.33	90.81 ± 5.08	88.23 ± 5.62	87.65 ± 5.46	87.44 ± 5.96	85.12 ± 6.82
		KNN	92.84 ± 3.67	91.39 ± 3.59	89.58 ± 5.67	87.07 ± 6.37	86.35 ± 6.57	86.44 ± 6.99	83.39 ± 7.65
Training and testing with high force	Accuracy	LDA	97.44 ± 1.10	96.69 ± 1.60	96.34 ± 1.75	95.56 ± 1.70	95.40 ± 1.98	95.69 ± 1.50	94.89 ± 1.88
		SVM	97.32 ± 1.22	96.63 ± 1.67	96.32 ± 1.70	95.47 ± 1.77	95.36 ± 1.94	95.52 ± 1.50	94.89 ± 1.99
		KNN	97.13 ± 1.25	96.44 ± 1.86	95.95 ± 1.90	95.10 ± 1.85	94.99 ± 2.16	95.07 ± 1.75	94.39 ± 2.17
	Sensitivity	LDA	92.33 ± 3.30	90.07 ± 4.80	89.01 ± 5.24	86.69 ± 5.09	86.20 ± 5.94	87.07 ± 4.50	84.68 ± 5.63
		SVM	91.97 ± 3.66	89.90 ± 5.01	88.96 ± 5.09	86.41 ± 5.31	86.08 ± 5.81	86.57 ± 4.50	84.68 ± 5.97
		KNN	91.38 ± 3.75	89.31 ± 5.57	87.84 ± 5.69	85.30 ± 5.55	84.97 ± 6.49	85.21 ± 5.24	83.18 ± 6.51
	Specificity	LDA	98.54 ± 0.65	98.11 ± 0.91	97.90 ± 1.01	97.43 ± 0.99	97.33 ± 1.17	97.52 ± 0.92	97.06 ± 1.14
		SVM	98.48 ± 0.71	98.07 ± 0.97	97.90 ± 0.99	97.38 ± 1.03	97.31 ± 1.15	97.41 ± 0.92	97.07 ± 1.20
		KNN	98.36 ± 0.72	97.94 ± 1.09	97.67 ± 1.12	97.14 ± 1.10	97.05 ± 1.32	97.13 ± 1.09	96.73 ± 1.33
	Precision	LDA	92.97 ± 3.11	90.97 ± 4.29	90.11 ± 4.21	87.74 ± 4.73	87.35 ± 5.15	87.98 ± 4.38	86.06 ± 4.90
		SVM	92.79 ± 3.52	90.79 ± 4.61	90.03 ± 4.13	87.51 ± 4.93	87.28 ± 5.06	87.46 ± 4.26	86.01 ± 5.36
		KNN	92.24 ± 3.52	90.24 ± 5.11	88.93 ± 4.80	86.31 ± 5.25	86.09 ± 6.01	86.25 ± 5.06	84.48 ± 5.95
	F1 Score	LDA	92.07 ± 3.40	89.77 ± 4.87	88.67 ± 5.12	86.37 ± 5.03	85.78 ± 5.87	86.75 ± 4.45	84.23 ± 5.53
		SVM	91.67 ± 3.78	89.58 ± 5.08	88.61 ± 4.94	86.13 ± 5.28	85.70 ± 5.80	86.23 ± 4.41	84.26 ± 5.94
		KNN	91.09 ± 3.84	88.96 ± 5.72	87.47 ± 5.61	84.94 ± 5.54	84.61 ± 6.46	84.89 ± 5.16	82.74 ± 6.53

**Table A2:** The EMG pattern recognition performances when the classifiers are trained with a single force level and tested with all force levels.

	Parameter	Classifier	Proposed	TSD	TDPSD	Wavelet	TDF	AR-RMS	TD
Training with low force	Accuracy	LDA	89.07 ± 3.15	88.04 ± 2.61	88.48 ± 2.78	87.63 ± 2.91	86.52 ± 3.04	87.23 ± 2.71	86.18 ± 2.92
		SVM	89.10 ± 2.79	88.01 ± 2.57	88.57 ± 2.73	87.70 ± 2.83	86.46 ± 3.00	87.28 ± 2.94	86.15 ± 2.87
		KNN	89.02 ± 2.82	88.06 ± 2.64	88.55 ± 2.64	87.57 ± 2.86	86.84 ± 2.82	87.27 ± 2.84	86.50 ± 2.70
	Sensitivity	LDA	67.22 ± 9.44	64.13 ± 7.84	65.43 ± 8.33	62.90 ± 8.74	59.57 ± 9.13	61.69 ± 8.12	58.55 ± 8.76
		SVM	67.29 ± 8.38	64.03 ± 7.71	65.72 ± 8.19	63.11 ± 8.49	59.37 ± 8.99	61.83 ± 8.82	58.46 ± 8.60
		KNN	67.07 ± 8.45	64.17 ± 7.91	65.64 ± 7.92	62.72 ± 8.58	60.53 ± 8.45	61.81 ± 8.51	59.50 ± 8.11
	Specificity	LDA	93.72 ± 1.75	93.11 ± 1.49	93.34 ± 1.59	92.87 ± 1.68	92.17 ± 1.77	92.59 ± 1.58	92.00 ± 1.69
		SVM	93.70 ± 1.64	93.11 ± 1.47	93.4 ± 1.53	92.91 ± 1.71	92.07 ± 1.85	92.62 ± 1.75	91.87 ± 1.75
		KNN	93.65 ± 1.65	93.12 ± 1.54	93.36 ± 1.5	92.82 ± 1.69	92.30 ± 1.70	92.59 ± 1.76	92.11 ± 1.69
	Precision	LDA	75.51 ± 5.90	72.35 ± 4.97	73.37 ± 7.31	70.74 ± 6.72	67.70 ± 7.93	69.21 ± 6.65	66.75 ± 7.87
		SVM	74.63 ± 5.92	72.21 ± 5.51	73.21 ± 6.62	70.49 ± 6.57	67.55 ± 7.98	69.06 ± 7.45	66.55 ± 8.55
		KNN	74.21 ± 5.94	72.06 ± 5.85	72.81 ± 6.96	69.84 ± 7.07	66.83 ± 7.42	67.49 ± 7.71	65.42 ± 8.22
	F1 Score	LDA	67.04 ± 9.07	64.00 ± 7.31	65.10 ± 7.95	62.58 ± 8.48	59.23 ± 8.76	61.11 ± 7.90	58.05 ± 8.48
		SVM	67.22 ± 8.12	64.02 ± 7.17	65.40 ± 8.05	62.85 ± 8.21	59.40 ± 8.49	61.52 ± 8.54	58.30 ± 8.30
		KNN	67.07 ± 8.19	64.22 ± 7.41	65.35 ± 7.86	62.47 ± 8.39	60.16 ± 8.14	61.31 ± 8.31	58.96 ± 8.11
***Training with medium force***	Accuracy	LDA	91.99 ± 2.35	90.86 ± 2.05	90.66 ± 2.63	90.57 ± 2.41	89.20 ± 2.97	90.03 ± 2.40	88.96 ± 3.03
		SVM	91.94 ± 2.44	90.82 ± 2.07	90.78 ± 2.56	90.45 ± 2.39	89.26 ± 2.82	89.91 ± 2.49	88.78 ± 2.90
		KNN	91.89 ± 2.42	90.86 ± 1.92	90.76 ± 2.67	90.27 ± 2.56	89.22 ± 3.06	89.81 ± 2.65	88.76 ± 2.92
	Sensitivity	LDA	75.97 ± 7.06	72.58 ± 6.14	71.97 ± 7.88	71.70 ± 7.22	67.61 ± 8.91	70.10 ± 7.20	66.89 ± 9.09
		SVM	75.81 ± 7.33	72.46 ± 6.21	72.35 ± 7.68	71.34 ± 7.17	67.79 ± 8.47	69.74 ± 7.46	66.34 ± 8.70
		KNN	75.67 ± 7.26	72.57 ± 5.77	72.27 ± 8.00	70.80 ± 7.68	67.67 ± 9.19	69.44 ± 7.95	66.29 ± 8.76
	Specificity	LDA	95.29 ± 1.44	94.61 ± 1.30	94.49 ± 1.59	94.41 ± 1.49	93.55 ± 1.81	94.11 ± 1.44	93.38 ± 1.85
		SVM	95.24 ± 1.50	94.57 ± 1.31	94.54 ± 1.57	94.34 ± 1.49	93.56 ± 1.74	94.02 ± 1.50	93.26 ± 1.78
		KNN	95.21 ± 1.50	94.58 ± 1.23	94.52 ± 1.65	94.22 ± 1.61	93.52 ± 1.91	93.95 ± 1.62	93.23 ± 1.83
	Precision	LDA	78.70 ± 5.93	75.77 ± 4.91	75.12 ± 6.73	74.03 ± 6.54	70.41 ± 8.26	72.37 ± 7.12	69.18 ± 8.73
		SVM	78.57 ± 6.02	75.71 ± 4.76	75.05 ± 6.94	73.45 ± 6.49	70.63 ± 7.90	72.03 ± 7.17	69.03 ± 8.24
		KNN	78.27 ± 6.10	75.55 ± 4.51	74.69 ± 7.39	72.77 ± 7.06	70.11 ± 8.51	71.31 ± 8.17	68.33 ± 8.51
	F1 Score	LDA	75.83 ± 6.89	72.47 ± 5.94	71.75 ± 7.53	71.44 ± 6.92	67.42 ± 8.68	69.94 ± 7.08	66.64 ± 8.90
		SVM	75.76 ± 7.08	72.46 ± 5.85	72.13 ± 7.55	71.09 ± 6.91	67.61 ± 8.18	69.61 ± 7.34	66.09 ± 8.46
		KNN	75.61 ± 7.05	72.54 ± 5.48	72.08 ± 7.78	70.53 ± 7.47	67.52 ± 8.95	69.20 ± 7.98	66.01 ± 8.70
Training with high force	Accuracy	LDA	89.93 ± 2.26	88.76 ± 2.21	88.31 ± 2.70	88.11 ± 2.29	87.20 ± 2.47	87.92 ± 2.65	86.24 ± 2.90
		SVM	89.72 ± 2.11	88.65 ± 2.03	88.21 ± 2.61	88.04 ± 2.10	87.32 ± 2.47	87.76 ± 2.44	86.40 ± 2.73
		KNN	89.53 ± 2.38	88.47 ± 2.16	87.95 ± 2.76	87.55 ± 2.18	86.76 ± 2.55	87.19 ± 2.46	85.80 ± 2.63
	Sensitivity	LDA	69.79 ± 6.79	66.28 ± 6.64	64.93 ± 8.09	64.32 ± 6.88	61.60 ± 7.42	63.76 ± 7.94	58.73 ± 8.70
		SVM	69.17 ± 6.33	65.94 ± 6.09	64.64 ± 7.84	64.12 ± 6.29	61.95 ± 7.42	63.28 ± 7.32	59.21 ± 8.19
		KNN	68.60 ± 7.13	65.42 ± 6.47	63.84 ± 8.27	62.64 ± 6.55	60.29 ± 7.66	61.57 ± 7.39	57.40 ± 7.88
	Specificity	LDA	93.90 ± 1.32	93.18 ± 1.29	92.85 ± 1.63	92.75 ± 1.34	92.11 ± 1.57	92.61 ± 1.57	91.56 ± 1.69
		SVM	93.77 ± 1.23	93.10 ± 1.20	92.76 ± 1.57	92.67 ± 1.28	92.12 ± 1.58	92.50 ± 1.46	91.59 ± 1.65
		KNN	93.63 ± 1.41	92.97 ± 1.31	92.54 ± 1.68	92.35 ± 1.32	91.78 ± 1.66	92.12 ± 1.49	91.22 ± 1.60
	Precision	LDA	74.84 ± 5.30	72.18 ± 4.89	70.51 ± 7.23	69.12 ± 6.66	66.68 ± 7.46	68.12 ± 7.14	63.70 ± 8.82
		SVM	74.36 ± 4.89	71.89 ± 4.11	70.85 ± 6.47	68.84 ± 6.32	66.73 ± 7.20	68.17 ± 6.58	65.00 ± 8.29
		KNN	73.82 ± 5.34	71.18 ± 5.10	70.60 ± 7.21	67.79 ± 6.46	66.36 ± 8.11	66.38 ± 7.18	63.85 ± 8.82
	F1 Score	LDA	69.10 ± 6.63	65.67 ± 6.42	63.63 ± 8.51	63.35 ± 7.15	60.46 ± 7.70	62.99 ± 8.17	57.69 ± 8.79
		SVM	68.43 ± 5.93	65.27 ± 5.57	63.43 ± 8.12	63.16 ± 6.51	60.99 ± 7.65	62.58 ± 7.43	58.42 ± 8.30
		KNN	68.05 ± 6.80	64.86 ± 6.21	62.72 ± 8.54	61.80 ± 6.84	59.23 ± 7.91	60.77 ± 7.65	56.60 ± 8.07

**Table A3:** The EMG pattern recognition performances when the classifiers are trained with two force levels and tested with all force levels.

	Parameter	Classifier	Proposed	TSD	TDPSD	Wavelet	TDF	AR-RMS	TD
Training with low and medium forces	Accuracy	LDA	94.21 ± 1.83	93.30 ± 1.66	93.06 ± 2.34	92.80 ± 2.23	91.53 ± 2.69	92.06 ± 1.86	91.05 ± 2.52
		SVM	94.20 ± 1.84	93.22 ± 1.63	93.12 ± 2.32	92.75 ± 2.26	91.76 ± 2.59	92.02 ± 2.16	91.12 ± 2.82
		KNN	93.90 ± 1.97	93.03 ± 1.74	92.85 ± 2.47	92.37 ± 2.46	91.49 ± 2.81	91.72 ± 2.28	90.86 ± 2.90
	Sensitivity	LDA	82.64 ± 5.50	79.90 ± 4.97	79.18 ± 7.03	78.40 ± 6.70	74.60 ± 8.07	76.18 ± 5.59	73.14 ± 7.57
		SVM	82.60 ± 5.52	79.66 ± 4.89	79.37 ± 6.95	78.24 ± 6.79	75.28 ± 7.76	76.06 ± 6.48	73.36 ± 8.45
		KNN	81.70 ± 5.90	79.09 ± 5.22	78.54 ± 7.42	77.11 ± 7.38	74.47 ± 8.44	75.15 ± 6.85	72.57 ± 8.69
	Specificity	LDA	96.64 ± 1.05	96.07 ± 0.95	95.95 ± 1.37	95.79 ± 1.31	95.00 ± 1.60	95.32 ± 1.11	94.68 ± 1.50
		SVM	96.63 ± 1.06	96.03 ± 0.92	95.97 ± 1.35	95.75 ± 1.34	95.12 ± 1.52	95.30 ± 1.26	94.72 ± 1.67
		KNN	96.44 ± 1.14	95.90 ± 1.01	95.80 ± 1.46	95.51 ± 1.48	94.93 ± 1.71	95.09 ± 1.39	94.53 ± 1.76
	Precision	LDA	84.45 ± 4.98	81.95 ± 4.49	81.28 ± 6.79	80.19 ± 6.37	76.89 ± 7.89	77.98 ± 5.72	75.26 ± 7.81
		SVM	84.37 ± 4.96	81.8 ± 4.27	81.28 ± 6.70	80.03 ± 6.43	77.24 ± 7.44	77.90 ± 6.18	75.55 ± 8.14
		KNN	83.29 ± 5.59	80.98 ± 4.93	80.24 ± 7.34	78.53 ± 7.41	75.91 ± 8.58	76.34 ± 7.18	73.94 ± 9.00
	F1 Score	LDA	82.63 ± 5.40	79.91 ± 4.92	79.13 ± 6.96	78.30 ± 6.59	74.64 ± 7.85	76.11 ± 5.58	73.04 ± 7.57
		SVM	82.60 ± 5.39	79.72 ± 4.75	79.36 ± 6.85	78.21 ± 6.65	75.26 ± 7.62	76.05 ± 6.36	73.34 ± 8.35
		KNN	81.69 ± 5.83	79.10 ± 5.16	78.49 ± 7.37	77.02 ± 7.35	74.38 ± 8.38	75.02 ± 6.90	72.32 ± 8.85
***Training with low and high forces***	Accuracy	LDA	95.34 ± 1.70	94.80 ± 1.61	94.17 ± 2.12	93.39 ± 2.12	92.55 ± 2.64	92.81 ± 2.24	91.57 ± 2.86
		SVM	95.37 ± 1.78	94.80 ± 1.70	94.25 ± 2.16	93.30 ± 2.17	92.49 ± 2.74	92.77 ± 2.35	91.60 ± 2.89
		KNN	94.98 ± 1.92	94.41 ± 1.87	93.72 ± 2.40	92.58 ± 2.48	91.77 ± 2.95	91.92 ± 2.60	90.73 ± 3.16
	Sensitivity	LDA	86.03 ± 5.10	84.41 ± 4.82	82.52 ± 6.35	80.18 ± 6.35	77.66 ± 7.93	78.43 ± 6.71	74.72 ± 8.58
		SVM	86.12 ± 5.34	84.39 ± 5.11	82.74 ± 6.49	79.89 ± 6.52	77.47 ± 8.23	78.31 ± 7.06	74.81 ± 8.68
		KNN	84.94 ± 5.75	83.23 ± 5.60	81.16 ± 7.21	77.74 ± 7.43	75.32 ± 8.84	75.75 ± 7.79	72.20 ± 9.47
	Specificity	LDA	97.25 ± 1.00	96.90 ± 0.95	96.55 ± 1.24	96.07 ± 1.24	95.53 ± 1.61	95.67 ± 1.34	94.94 ± 1.71
		SVM	97.26 ± 1.07	96.90 ± 1.02	96.58 ± 1.26	96.01 ± 1.29	95.49 ± 1.68	95.65 ± 1.42	94.97 ± 1.74
		KNN	97.02 ± 1.16	96.66 ± 1.12	96.25 ± 1.42	95.57 ± 1.50	95.03 ± 1.83	95.12 ± 1.59	94.41 ± 1.93
	Precision	LDA	86.81 ± 5.11	85.22 ± 4.78	83.61 ± 6.29	81.16 ± 6.36	78.61 ± 8.04	79.39 ± 6.60	76.00 ± 8.62
		SVM	86.92 ± 5.37	85.26 ± 5.03	83.85 ± 6.31	80.84 ± 6.55	78.54 ± 8.25	79.33 ± 6.91	76.15 ± 8.63
		KNN	85.69 ± 5.82	84.00 ± 5.73	82.11 ± 7.18	78.63 ± 7.55	76.30 ± 9.08	76.61 ± 7.81	73.37 ± 9.63
	F1 Score	LDA	85.92 ± 5.17	84.27 ± 4.90	82.33 ± 6.43	80.06 ± 6.39	77.49 ± 8.02	78.31 ± 6.75	74.51 ± 8.74
		SVM	86.05 ± 5.38	84.31 ± 5.15	82.62 ± 6.52	79.82 ± 6.53	77.43 ± 8.23	78.27 ± 7.06	74.79 ± 8.72
		KNN	84.84 ± 5.84	83.11 ± 5.72	81.02 ± 7.26	77.63 ± 7.52	75.21 ± 8.95	75.64 ± 7.89	72.08 ± 9.63
Training with medium and high forces	Accuracy	LDA	94.27 ± 1.83	93.20 ± 1.54	93.00 ± 2.23	92.57 ± 2.24	91.71 ± 2.87	92.06 ± 2.29	90.82 ± 2.78
		SVM	94.18 ± 1.86	93.24 ± 1.49	93.16 ± 2.30	92.45 ± 2.29	91.95 ± 2.88	91.92 ± 2.29	91.11 ± 2.72
		KNN	93.88 ± 1.91	92.95 ± 1.60	92.82 ± 2.34	91.97 ± 2.43	91.24 ± 3.06	91.23 ± 2.45	90.29 ± 2.93
	Sensitivity	LDA	82.81 ± 5.50	79.60 ± 4.61	79.01 ± 6.70	77.72 ± 6.71	75.14 ± 8.62	76.18 ± 6.86	72.46 ± 8.33
		SVM	82.53 ± 5.58	79.71 ± 4.48	79.49 ± 6.91	77.34 ± 6.87	75.86 ± 8.64	75.75 ± 6.87	73.33 ± 8.15
		KNN	81.64 ± 5.72	78.86 ± 4.80	78.46 ± 7.01	75.91 ± 7.30	73.71 ± 9.18	73.68 ± 7.35	70.87 ± 8.78
	Specificity	LDA	96.62 ± 1.09	95.95 ± 0.94	95.83 ± 1.33	95.55 ± 1.38	95.00 ± 1.80	95.24 ± 1.37	94.44 ± 1.72
		SVM	96.55 ± 1.11	95.97 ± 0.91	95.92 ± 1.39	95.48 ± 1.41	95.13 ± 1.79	95.16 ± 1.38	94.61 ± 1.69
		KNN	96.37 ± 1.15	95.79 ± 0.99	95.70 ± 1.42	95.18 ± 1.50	94.68 ± 1.93	94.71 ± 1.49	94.09 ± 1.83
	Precision	LDA	84.54 ± 4.81	81.72 ± 3.94	80.95 ± 6.42	79.06 ± 6.72	76.90 ± 8.29	77.50 ± 6.83	74.29 ± 8.25
		SVM	84.38 ± 4.70	81.79 ± 3.66	81.37 ± 6.51	78.77 ± 6.64	77.42 ± 8.33	77.17 ± 6.82	74.92 ± 8.24
		KNN	83.43 ± 4.94	80.95 ± 4.05	80.12 ± 6.81	77.19 ± 7.17	75.29 ± 9.02	74.93 ± 7.62	72.51 ± 8.95
	F1 Score	LDA	82.65 ± 5.47	79.42 ± 4.58	78.65 ± 6.85	77.41 ± 6.85	74.71 ± 8.75	75.86 ± 7.18	72.07 ± 8.49
		SVM	82.40 ± 5.50	79.57 ± 4.41	79.18 ± 6.99	77.10 ± 6.92	75.49 ± 8.72	75.48 ± 7.09	72.96 ± 8.36
		KNN	81.54 ± 5.61	78.71 ± 4.77	78.10 ± 7.13	75.64 ± 7.37	73.36 ± 9.32	73.34 ± 7.66	70.47 ± 9.07

**Table A4:** The EMG pattern recognition performances when the classifiers are trained and tested with all force levels.

Parameter	Classifier	Proposed	TSD	TDPSD	Wavelet	TDF	AR-RMS	TD
Accuracy	LDA	96.30 ± 1.52	95.75 ± 1.38	95.28 ± 1.94	94.45 ± 2.03	93.69 ± 2.60	93.89 ± 2.02	92.71 ± 2.74
	SVM	96.37 ± 1.60	95.80 ± 1.42	95.34 ± 2.03	94.37 ± 2.14	93.78 ± 2.62	93.98 ± 2.09	92.91 ± 2.77
	KNN	95.88 ± 1.77	95.33 ± 1.68	94.79 ± 2.28	93.62 ± 2.51	92.95 ± 2.99	93.07 ± 2.48	91.88 ± 3.10
Sensitivity	LDA	88.89 ± 4.55	87.24 ± 4.13	85.83 ± 5.81	83.34 ± 6.10	81.07 ± 7.81	81.68 ± 6.06	78.12 ± 8.22
	SVM	89.11 ± 4.80	87.41 ± 4.25	86.02 ± 6.09	83.10 ± 6.43	81.35 ± 7.87	81.93 ± 6.28	78.72 ± 8.30
	KNN	87.63 ± 5.32	86.00 ± 5.04	84.36 ± 6.85	80.86 ± 7.52	78.84 ± 8.96	79.21 ± 7.43	75.65 ± 9.31
Specificity	LDA	97.82 ± 0.90	97.51 ± 0.81	97.22 ± 1.14	96.71 ± 1.23	96.24 ± 1.60	96.36 ± 1.22	95.63 ± 1.67
	SVM	97.86 ± 0.95	97.53 ± 0.83	97.26 ± 1.20	96.66 ± 1.29	96.29 ± 1.59	96.41 ± 1.27	95.77 ± 1.67
	KNN	97.56 ± 1.07	97.24 ± 1.01	96.91 ± 1.35	96.19 ± 1.54	95.76 ± 1.85	95.84 ± 1.52	95.11 ± 1.90
Precision	LDA	89.31 ± 4.50	87.85 ± 4.04	86.50 ± 5.79	83.86 ± 6.16	81.59 ± 8.02	82.20 ± 6.19	78.79 ± 8.44
	SVM	89.54 ± 4.71	88.01 ± 4.13	86.71 ± 5.96	83.62 ± 6.46	81.90 ± 7.86	82.43 ± 6.31	79.36 ± 8.34
	KNN	88.01 ± 5.31	86.51 ± 5.04	84.92 ± 6.86	81.21 ± 7.73	79.32 ± 9.12	79.56 ± 7.64	76.19 ± 9.59
F1 Score	LDA	88.81 ± 4.58	87.16 ± 4.17	85.69 ± 5.85	83.20 ± 6.16	80.86 ± 7.91	81.54 ± 6.15	77.90 ± 8.31
	SVM	89.06 ± 4.81	87.36 ± 4.27	85.93 ± 6.09	83.00 ± 6.44	81.23 ± 7.89	81.85 ± 6.33	78.59 ± 8.35
	KNN	87.56 ± 5.37	85.93 ± 5.09	84.24 ± 6.89	80.69 ± 7.64	78.67 ± 9.09	79.04 ± 7.58	75.44 ± 9.50
